# Ten-Year Trends in Serum 25-Hydroxyvitamin D in Slovenia (2014–2023): Laboratory-Based Data from Tested Individuals and COVID-19-Period Changes

**DOI:** 10.3390/nu18071168

**Published:** 2026-04-07

**Authors:** Joško Osredkar, Darko Siuka, Aleš Jerin, Borut Štabuc, Uroš Godnov

**Affiliations:** 1 Institute of Clinical Chemistry and Biochemistry, University Medical Centre Ljubljana, Zaloška Cesta 2, 1000 Ljubljana, Slovenia; josko.osredkar@kclj.si (J.O.);; 2 Faculty of Pharmacy, University of Ljubljana, Aškerčeva 7, 1000 Ljubljana, Slovenia; 3 Department of Gastroenterology, University Medical Centre Ljubljana, Zaloška Cesta 2, 1000 Ljubljana, Slovenia; darko.siuka@kclj.si (D.S.); borut.stabuc@kclj.si (B.Š.); 4 Faculty of Mathematics, Natural Science and Information Technologies, University of Primorska, Glagoljaška Ulica 8, 6000 Koper, Slovenia

**Keywords:** 25-hydroxyvitamin D, seasonal variation, deficiency, age factors, COVID-19 pandemic

## Abstract

Background: Vitamin D status is influenced by season, age, and public health messaging. The COVID-19 pandemic was accompanied by heightened interest in vitamin D, but long-term national data from Central/Eastern Europe remain limited. We aimed to characterize 10-year trends, seasonal variation, and demographic determinants of serum 25-hydroxyvitamin D [25(OH)D] in Slovenia, with particular focus on changes during the COVID-19 period. Methods: We performed a retrospective cross-sectional analysis of all serum 25(OH)D measurements performed at the Slovenian national reference laboratory between January 2014 and December 2023. The core analytic cohort included 106,875 patients with valid 25(OH)D results, aged 0–100 years. Vitamin D status was classified as deficient (<30 nmol/L), insufficient (30–50 nmol/L), adequate (50–75 nmol/L), and optimal (>75 nmol/L). Temporal trends, seasonal patterns, and age- and sex-specific differences were assessed using non-parametric tests and Kendall’s τ. Results: Mean 25(OH)D concentration over the study period was 61.9 ± 34.2 nmol/L; 16.0% of patients were deficient and 22.8% insufficient. Annual mean 25(OH)D increased from 57.0 nmol/L in 2014 to 67.2 nmol/L in 2023, with a significant upward temporal trend and a 14.6% higher mean level during 2020–2023 compared with 2014–2019. Seasonal variation was pronounced (≈20% higher summer–autumn vs. winter–spring), and vitamin D status declined progressively with age, with the highest deficiency prevalence in patients ≥ 70 years. Females had slightly higher 25(OH)D than males, although absolute differences were small. Conclusions: This laboratory-based analysis of tested patients showed higher 25(OH)D concentrations during and after the COVID-19 period, superimposed on persistent seasonal and age-related gradients. These observations identify older adults and winter testing periods as important contexts for vitamin D optimization, but they should be interpreted as descriptive trends among tested individuals rather than as evidence of causal pandemic effects or population-wide prevalence changes.

## 1. Introduction

Vitamin D, a fat-soluble secosteroid, plays essential roles beyond calcium homeostasis and bone health, including regulation of immune function, cellular proliferation, and cardiovascular health [1,2]. Emerging evidence highlights that 25(OH)D serves primarily as a circulating substrate that is locally converted to 1,25-dihydroxyvitamin D within target tissues in proportion to its availability, largely independent of parathyroid hormone, thereby supporting a broader set of extraskeletal actions [3]. The primary source of vitamin D in humans is cutaneous synthesis through ultraviolet B (UVB) radiation exposure, though dietary intake and supplementation contribute to overall status [4]. Serum 25-hydroxyvitamin D [25(OH)D] concentration serves as the most reliable biomarker for vitamin D status due to its relatively long half-life of approximately 2–3 weeks and reflection of both endogenous synthesis and exogenous intake [5].

Vitamin D deficiency and insufficiency remain highly prevalent worldwide, affecting an estimated 1 billion people globally [6]. Suboptimal vitamin D status has been associated with increased risk of osteoporosis, fractures, autoimmune diseases, infectious diseases, cardiovascular disorders, and certain malignancies [7,8,9]. Geographic latitude, season, age, skin pigmentation, lifestyle factors, and dietary habits all influence vitamin D status, creating complex patterns of deficiency across populations [10,11].

The marked seasonal variation in serum 25(OH)D concentrations, particularly in temperate and northern latitudes, reflects the angle of sun exposure and consequent changes in UVB radiation availability [12]. During winter months at higher latitudes, UVB photons are insufficient for cutaneous vitamin D synthesis, leading to predictable declines in serum 25(OH)D concentrations [13]. This seasonal oscillation has important clinical implications, as winter deficiency may contribute to increased susceptibility to respiratory infections and exacerbation of chronic conditions [14,15].

Age significantly influences vitamin D status through multiple mechanisms. Elderly individuals demonstrate reduced capacity for cutaneous vitamin D synthesis, decreased renal conversion of 25(OH)D to its active form 1,25-dihydroxyvitamin D, reduced outdoor activity, and often inadequate dietary intake [16,17]. Additionally, age-related changes in body composition, decreased intestinal absorption efficiency, and concurrent medications may further compromise vitamin D status in older adults [18,19]. Understanding age-specific patterns of vitamin D deficiency is crucial for developing targeted intervention strategies.

The COVID-19 pandemic, which began in early 2020, profoundly impacted public health awareness and behaviors related to vitamin D. Initial observational studies suggested potential associations between vitamin D deficiency and COVID-19 severity, prompting widespread media attention and increased public interest in supplementation [20,21]. Several mechanisms were proposed, including vitamin D’s role in modulating innate and adaptive immune responses, regulating the renin-angiotensin system, and potentially influencing viral entry and replication [22,23]. Ecological analyses from European countries suggested a strong correlation between the prevalence of severe vitamin D deficiency and COVID-19 mortality rates, further amplifying public and scientific interest in the potential protective role of adequate vitamin D status during the pandemic [24]. Although subsequent randomized controlled trials yielded mixed results regarding vitamin D supplementation for COVID-19 prevention or treatment [25,26], the heightened public awareness may have influenced supplementation behaviors and clinical testing practices.

The pharmacokinetics of vitamin D supplementation are important to consider when interpreting temporal trends. Following oral or intramuscular administration, vitamin D2 or D3 undergoes hepatic 25-hydroxylation to form 25(OH)D, the major circulating metabolite that reflects both stored and recently absorbed vitamin D. The half-life of 25(OH)D is approximately 2–3 weeks, meaning that changes in supplementation practices would be reflected in serum levels within 3–6 weeks, with stable new steady-state levels achieved within 8–12 weeks [27]. This pharmacokinetic window is critical for interpreting the observed pandemic-era changes, as behavioral modifications following initial COVID-19 awareness in March 2020 would be expected to manifest as detectable 25(OH)D changes by mid-to-late 2020, consistent with our observations.

Despite extensive research on vitamin D, large-scale, long-term studies examining temporal trends in vitamin D status across diverse age groups and spanning the COVID-19 pandemic remain limited. Most existing studies focus on specific populations or shorter timeframes, limiting generalizability and the ability to detect population-level changes in response to public health events [28,29].

The primary aim of this study was to characterize temporal trends in serum 25(OH)D concentrations from 2014 to 2023, with particular focus on changes during the COVID-19 pandemic period. Secondary aims included quantifying seasonal variation, identifying age-related and sex-related patterns, and determining the prevalence of vitamin D deficiency across these subgroups using data from 107,900 laboratory records, of which 106,875 patients with valid 25OHD measurements formed the core analytic cohort. These analyses were designed to characterize temporal and demographic patterns in serum 25(OH)D among individuals undergoing laboratory testing, rather than to estimate population-representative prevalence in Slovenia.

## 2. Methods and Materials

### 2.1. Study Design and Setting

This retrospective cross-sectional study analyzed serum 25-hydroxyvitamin D [25(OH)D] measurements from patients who underwent laboratory testing at Clinical Institute of Clinical Chemistry and Biochemistry, University Medical Center Ljubljana, between 2 January 2014 and 29 December 2023. Although the laboratory functions as a national reference center, the present dataset does not constitute a population-based sample. Accordingly, the term “nationwide” refers to the scope of the laboratory data source rather than to the representativeness of the general population. The study was conducted in accordance with the Declaration of Helsinki and was approved by the institutional ethics committee of University Medical Center Ljubljana (KSEV-5-111125; approval date 11 November 2025), which specifically authorized the retrospective use of anonymized laboratory data from the 2014–2023 period for the present study. Data extraction and analysis were performed only after this approval had been obtained. Due to the retrospective nature of the study and use of anonymized data, the requirement for informed consent was waived.

### 2.2. Study Population

All patients aged 0 years and older who had at least one serum 25(OH)D measurement during the study period were eligible for inclusion. Patients were included regardless of the clinical indication for vitamin D testing. Laboratory tests were ordered by physicians across various clinical departments, including pediatrics, internal medicine, endocrinology, geriatrics, oncology, and general practice. Requests originated from routine clinical care across multiple settings, including pediatrics, internal medicine, endocrinology, geriatrics, oncology, and general practice. However, the specific clinical indication for 25(OH)D testing was not recorded in a standardized and analyzable form in the laboratory information system. Accordingly, the present analysis cannot distinguish between tests ordered for suspected deficiency, osteoporosis, or metabolic bone disease assessment, malabsorption, chronic comorbidity follow-up, preventive evaluation, or acute illness.

Clinical indications for 25(OH)D testing (e.g., osteoporosis assessment, malabsorption, suspected deficiency, or acute illness, including COVID-19) were not uniformly recorded in the laboratory information system and therefore could not be analyzed. As a result, we were unable to differentiate vitamin D status according to specific diagnostic categories or to isolate tests ordered explicitly for COVID-19-related illness.

For patients with multiple 25(OH)D measurements during the study period, only the first measurement was included in the analyses to ensure statistical independence of observations. Exclusion criteria were minimal: patients with missing or implausible demographic data (date of birth or sex) were excluded. No restrictions were placed on concurrent medications, comorbidities, or vitamin D supplementation status.

Of the 107,900 initial records retrieved, data quality assessment identified systematic data entry errors affecting age and vitamin D concentration. Records with biologically implausible values were excluded: 180 records (0.17%) with age > 100 years (maximum 119.8 years, all with birth date 1 January 1900) and 45 records (0.04%) with 25(OH)D > 150 nmol/L (maximum 1356.5 nmol/L). Final analytical cohort: 106,875 participants (99.79%). Sensitivity analysis confirmed exclusions had a negligible impact on descriptive statistics.

### 2.3. Data Collection

Data were extracted from the laboratory information system using standardized query protocols. The following variables were collected: date of birth, sex, date of sample collection, serum 25(OH)D concentration, and concurrent biochemical variables when available (serum calcium, phosphate, albumin, alkaline phosphatase, creatinine). Age at sampling was calculated as the difference between the sample collection date and the date of birth, expressed in years. All extracted data were anonymized prior to analysis.

### 2.4. Laboratory Measurements

Serum 25-hydroxyvitamin D [25(OH)D] concentrations were measured using a competitive luminescence immunoassay (Architect analyzer, Abbott Diagnostics, Lake Forest, CA, USA) with a limit of quantification of 6 nmol/L as established during method validation and monitored throughout routine operation. This automated assay measures both 25(OH)D_2_ and 25(OH)D_3_ with high specificity and precision. All measurements were performed in the same ISO 15189-accredited central laboratory using the same assay platform throughout the study period, with continuous internal quality control and participation in external quality assessment schemes [30]. The long-term stability of internal CVs and external bias values makes major analytical drift an unlikely explanation for the observed temporal differences and modifications. The laboratory participates continuously in external quality assessment (EQA) schemes (e.g., INSTAND, DEQAS, and other IFCC-endorsed EQA schemes) and holds all required certificates for vitamin D measurement. Internal quality control for 25(OH)D is performed daily at three concentration levels. Over 2014–2023, long-term internal quality control (IQC) coefficients of variation ranged from 6.8% to 5.1% at the low control level (~30 nmol/L), 5.4% to 4.1% at the mid-level (~60 nmol/L), and 4.2% to 3.3% at the high control level (~80 nmol/L), showing progressive improvement in analytical precision (Supplementary Table S3). External quality assessment (Instand) results remained within ±10% of target values in >96% of survey samples throughout the study period, with bias consistently <±6% (Supplementary Table S3). This robust and stable quality control performance makes substantial analytical drift highly unlikely and supports the interpretation that observed temporal trends reflect genuine population-level changes in vitamin D status [31].

### 2.5. Variable Definitions

Vitamin D status was classified using thresholds aligned with Endocrine Society guidance and commonly used clinical categories in the literature, namely deficiency < 30 nmol/L, insufficiency 30–50 nmol/L, adequacy 50–75 nmol/L, and optimal status ≥ 75 nmol/L [32,33]. These thresholds were selected to facilitate comparison with prior clinical studies, although it should be acknowledged that cutoffs for vitamin D sufficiency remain debated and that alternative classifications could yield different prevalence estimates.

Age groups were defined as: 0–18 years (children/adolescents), 19–30 years (young adults), 31–40 years, 41–50 years, 51–60 years, 61–70 years, and ≥70 years (elderly).

Seasons were defined according to meteorological conventions: winter (December–February), spring (March–May), summer (June–August), and autumn (September–November).

The COVID period was classified as pre-COVID (1 January 2014 to 29 February 2020) versus COVID/post-COVID (1 March 2020 to 31 December 2023), with the cut-off corresponding to WHO’s pandemic declaration and implementation of public health measures [34].

### 2.6. Statistical Analysis

Continuous variables were assessed for normality using the Shapiro–Wilk test and visual inspection of Q-Q plots. Due to the right-skewed distribution of 25(OH)D concentrations, data are presented as both mean ± standard deviation and median with interquartile range (IQR). Categorical variables are presented as frequencies and percentages.

Two-group comparisons used Student’s *t*-test for normally distributed variables, Mann–Whitney U test for non-normally distributed variables, and chi-square test for categorical variables. Multiple group comparisons used one-way ANOVA or Kruskal–Wallis test, followed by post hoc pairwise comparisons using Dunn’s test with Bonferroni correction.

Spearman’s rank correlation coefficient (ρ) assessed relationships between continuous variables. Kendall’s tau (τ) assessed monotonic temporal trends. Correlation strength was interpreted as: negligible (|ρ| < 0.10), weak (0.10–0.39), moderate (0.40–0.69), strong (0.70–0.89), and very strong (≥0.90) [35].

All statistical tests were two-tailed, and *p*-values < 0.05 were considered statistically significant. Statistical analyses were performed using Python 3.12 with SciPy 1.11 and Pandas 2.0 libraries.

In addition to *p*-values, effect sizes and 95% confidence intervals were reported for major comparisons where appropriate, including odds ratios for categorical contrasts and standardized effect sizes for selected continuous-group comparisons.

### 2.7. Sample Size and Power

Of the 107,900 records initially retrieved, 225 (0.21%) were excluded because of biologically implausible values, yielding 107,675 cleaned records. Among these, 106,875 patients had valid 25(OH)D measurements and constituted the core analytic cohort used for all primary vitamin D analyses, tables, and figures. The remaining denominator differences reflect data availability for specific variables and are indicated where relevant. The maximum excluded vitamin D value (1356.5 nmol/L) was consistent with decimal point data entry error and clinically implausible. Sensitivity analysis confirmed that these exclusions had a negligible impact on descriptive statistics; mean 25(OH)D concentration (61.9 ± 34.2 nmol/L) and median concentration (59.0 nmol/L) remained unchanged before and after data cleaning, validating the robustness of the analytic dataset. For descriptive analyses of vitamin D status, we restricted the denominator to the 106,875 patients with valid 25(OH)D values. Minor differences in denominators across tables reflect whether analyses required only demographic data (107,675 cleaned records) or an available 25(OH)D measurement (106,875 records). These distinctions are explicitly indicated in each table. All primary analyses, tables, and figures are restricted to this core analytic cohort of 106,875 patients with valid 25(OH)D measurements.

Unless otherwise stated, all primary analyses, tables, and figures were conducted in the core analytic cohort of 106,875 patients with valid 25(OH)D measurements.

## 3. Results

### 3.1. Study Population and Baseline Characteristics

Between 2 January 2014 and 29 December 2023, 107,900 records with serum 25(OH)D testing were retrieved. After exclusion of 225 records with biologically implausible age or concentration values, 107,675 cleaned records remained. The core analytic cohort for primary analyses comprised 106,875 patients with valid 25(OH)D measurements (Table 1).

Overall mean 25(OH)D concentration was 61.9 ± 34.2 nmol/L, with median of 59.0 nmol/L (IQR: 39.0–79.0 nmol/L). Based on clinical thresholds, 17,059 patients (16.0%) had vitamin D deficiency, 24,354 (22.8%) had insufficiency, 35,096 (32.8%) had adequate levels, and 30,366 (28.4%) had optimal levels. Severe deficiency (<12.5 nmol/L) was observed in 1247 patients (1.2%).

The annual number of 25(OH)D measurements increased progressively from 6241 in 2014 to 14,621 in 2023. Distribution across seasons was relatively balanced: winter (28,049 patients, 26.0%), spring (28,717, 26.6%), summer (20,400, 18.9%), and autumn (30,734, 28.5%). The relative distribution of samples by season remained broadly stable across the study years, with only minor year-to-year fluctuations (Supplementary Table S1).

### 3.2. Temporal Trends and COVID-19 Pandemic Impact

**Figure 1:**
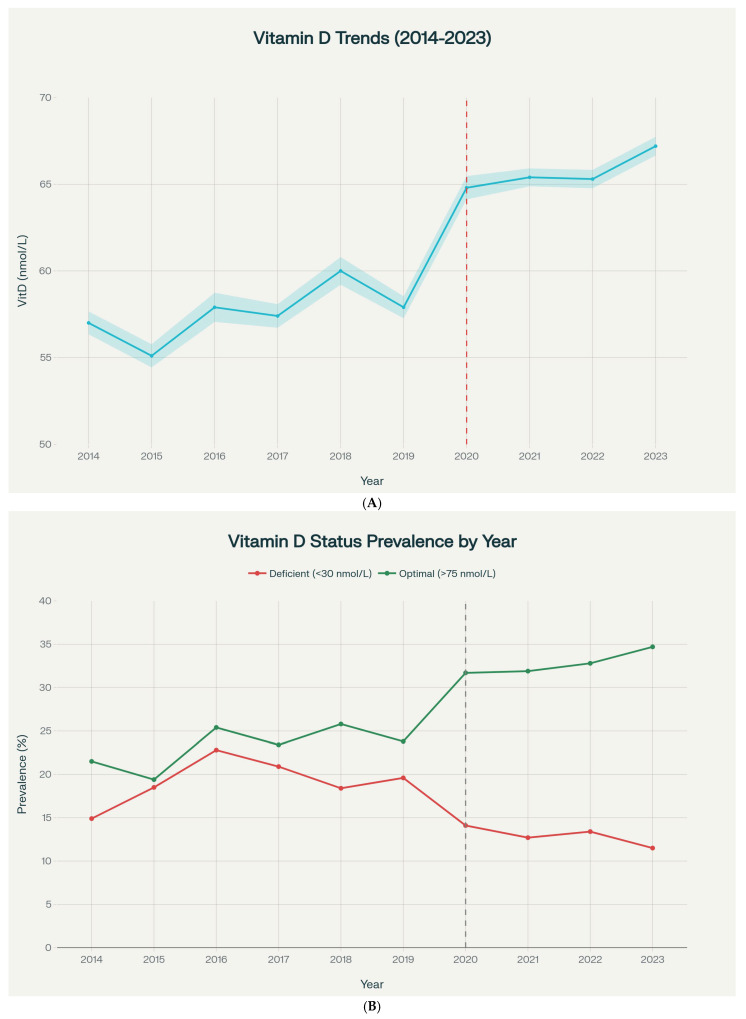
(**A**) Temporal trends in mean vitamin D levels from 2014 to 2023, showing a significant increase following the COVID-19 pandemic onset in 2020. All panels are based on the core analytic cohort of patients with valid 25(OH)D measurements (*n* = 106,875), unless otherwise indicated. (**B**) Prevalence of vitamin D deficiency and optimal status by year, showing declining deficiency rates and increasing optimal status after 2020. All panels are based on the core analytic cohort of patients with valid 25(OH)D measurements (*n* = 106,875), unless otherwise indicated.

Vitamin D levels demonstrated a significant upward temporal trend over the 10-year period (Kendall’s τ = 0.093, *p* < 0.001) (Supplementary Table S1, Figure 1A). Supplementary Table S1 presents the annual number of measurements, mean and median serum 25(OH)D concentrations, and the prevalence of deficiency and optimal status for each year from 2014 to 2023, providing detailed numerical values underlying the trends shown in Figure 1. Annual mean 25(OH)D increased from 57.0 ± 26.4 nmol/L in 2014 to 67.2 ± 33.2 nmol/L in 2023, representing a 10.2 nmol/L (17.9%) increase. Relatively stable levels were observed 2014–2019 (range: 55.1–60.0 nmol/L), followed by a marked increase beginning in 2020.

Deficiency prevalence fluctuated during pre-pandemic years (2014–2019: range 14.9–22.8%), but declined substantially from 2020 onward (2020–2023: range 11.5–14.1%) (Figure 1B). Conversely, optimal vitamin D status (>75 nmol/L) increased from approximately 20–26% in 2014–2019 to 32–35% in 2020–2023.

A notable finding was the sustained increase in vitamin D levels through 2023, three years after the initial pandemic onset. The mean 25(OH)D concentration in 2023 (67.2 ± 33.2 nmol/L) remained significantly increased compared to 2019 (57.9 ± 32.0 nmol/L), with no evidence of regression toward pre-pandemic baseline levels. Moreover, deficiency prevalence in 2023 (11.5%) was substantially lower than the 2019 baseline (19.6%), representing a sustained reduction of 41% relative to pre-pandemic rates. The persistence of higher 25(OH)D concentrations through 2023 indicates that the observed shift was not limited to the first pandemic year. However, the present dataset cannot determine whether this reflected sustained supplementation, altered healthcare utilization, changes in testing indication, differences in patient mix, or other unmeasured factors.

Between-period differences were interpreted using both statistical significance and effect-size magnitude, with emphasis on absolute differences in mean concentration and category prevalence rather than *p*-values alone (Table 2). Mean 25(OH)D increased from 57.6 ± 34.0 nmol/L pre-COVID to 66.0 ± 33.8 nmol/L during/post-COVID, representing 8.4 nmol/L increase (14.6%, Mann–Whitney U = 1.19 × 10^9^, *p* < 0.001). Median concentration similarly increased from 54.0 to 63.0 nmol/L (*p* < 0.001).

**Figure 2:**
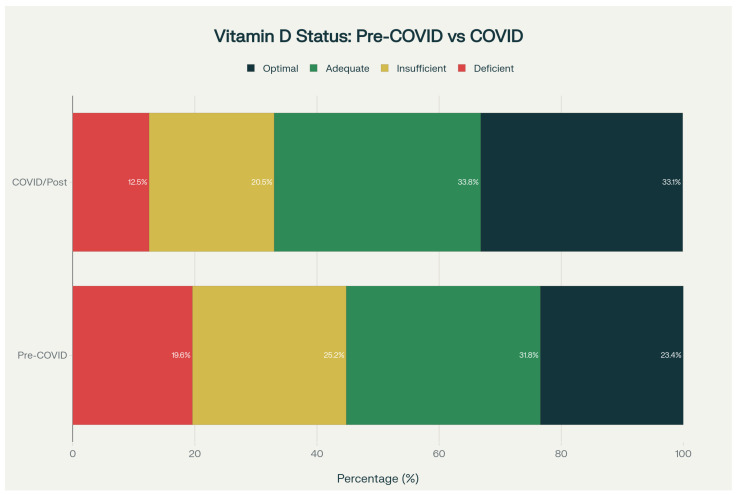
Distribution of vitamin D status in chronological order, comparing the pre-COVID period (2014–2019) with the COVID/post-COVID period (2020–2023), showing a shift toward higher 25(OH)D status categories in the later period (χ^2^ = 2023.58, *p* < 0.001). All panels are based on the core analytic cohort of patients with valid 25(OH)D measurements (*n* = 106,875), unless otherwise indicated.

Vitamin D status distribution shifted markedly between periods (χ^2^ = 2023.58, *df* = 3, *p* < 0.001) (Figure 2): deficiency decreased from 19.6% to 12.5%, insufficiency from 25.2% to 20.5%, while adequate status increased from 31.8% to 33.8%, and optimal status from 23.4% to 33.1%.

### 3.3. Seasonal Variation

**Figure 3:**
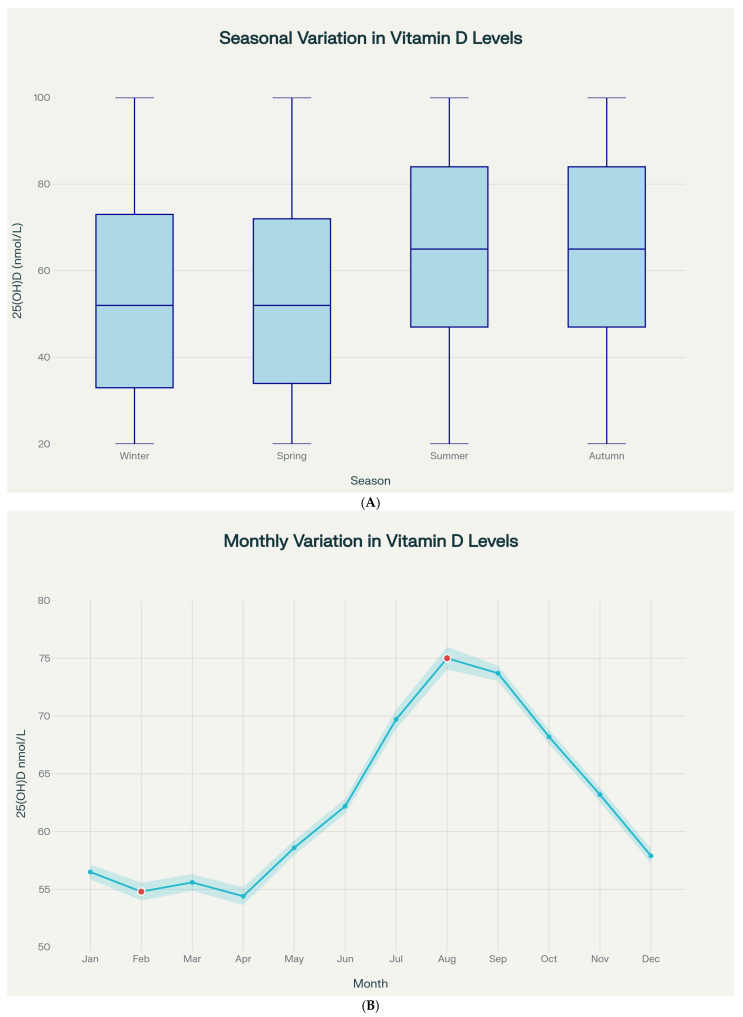
(**A**) Seasonal variation in vitamin D levels showing significantly higher concentrations in summer and autumn compared to winter and spring. All panels are based on the core analytic cohort of patients with valid 25(OH)D measurements (*n* = 106,875), unless otherwise indicated. (**B**) Monthly variation showing peak vitamin D levels in August (75.0 nmol/L) and lowest levels in February (54.8 nmol/L). All panels are based on the core analytic cohort of patients with valid 25(OH)D measurements (*n* = 106,875), unless otherwise indicated.

In the core analytic cohort of 106,875 patients with valid 25(OH)D measurements, pronounced seasonal variation in 25(OH)D was evident (Table 3, Figure 3A). Summer and autumn exhibited significantly higher levels compared to winter and spring (Kruskal–Wallis H = 4750.27, *df* = 3, *p* < 0.001): summer 68.0 ± 33.7 nmol/L (median: 65.0), autumn 68.1 ± 32.8 nmol/L (median: 65.0), winter 56.5 ± 33.1 nmol/L (median: 52.0), spring 56.3 ± 35.1 nmol/L (median: 52.0).

The difference between peak (summer/autumn) and nadir (winter/spring) was 11.8 nmol/L, representing 21% seasonal variation. Post hoc pairwise comparisons confirmed significant differences between all season pairs (*p* < 0.001 for all after Bonferroni correction).

Deficiency prevalence varied markedly by season: winter 21.2%, spring 21.0%, summer 10.4%, and autumn 10.2%, representing more than two-fold difference (χ^2^ = 2874.36, *df* = 3, *p* < 0.001).

Monthly analysis revealed August had the highest mean 25(OH)D (75.0 ± 37.2 nmol/L), while February showed the lowest (54.8 ± 35.2 nmol/L), yielding a peak-to-nadir difference of 20.2 nmol/L (36.9%) (Figure 3B). Supplementary Table S2 provides the full monthly distribution of serum 25(OH)D concentrations and deficiency prevalence for all 12 calendar months, complementing the graphical presentation in Figure 3B. Deficiency prevalence ranged from 24.7% (February) to 7.9% (September).

Seasonal patterns remained consistent in both pre-COVID and COVID/post-COVID periods, with no significant interaction (*p* = 0.412). However, absolute 25(OH)D concentrations were higher in the COVID/post-COVID period across all seasons.

### 3.4. Age-Related Differences

**Figure 4:**
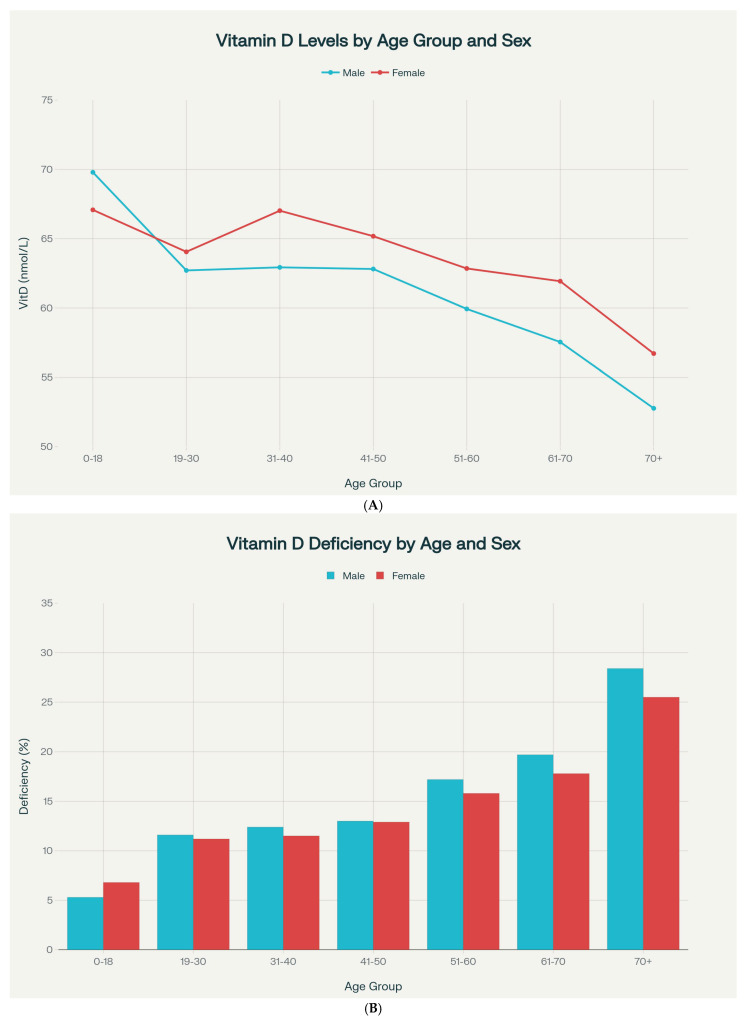
(**A**) Mean vitamin D levels across age groups stratified by sex, showing progressive decline with age in both males and females. All panels are based on the core analytic cohort of patients with valid 25(OH)D measurements (*n* = 106,875), unless otherwise indicated. (**B**) Prevalence of vitamin D deficiency increases with age in both sexes, from 5 to 7% in children to 25–28% in the elderly. All panels are based on the core analytic cohort of patients with valid 25(OH)D measurements (*n* = 106,875), unless otherwise indicated.

In the same core analytic cohort, vitamin D status demonstrated a significant inverse association with age (Spearman ρ = −0.179, *p* < 0.001). Analysis across seven age categories revealed progressive decline (Table 4, Figure 4A) (Kruskal–Wallis H = 2944.40, *df* = 6, *p* < 0.001).

The difference between the youngest and oldest age groups was 13.3 nmol/L, representing 19.4% decrease (Mann–Whitney U = 2.74 × 10^8^, *p* < 0.001, Cohen’s d = 0.40).

Deficiency prevalence increased markedly with age (Figure 4B) (χ^2^ for trend = 3856.72, *p* < 0.001): 0–18 years 6.1%, 19–30 years 11.4%, 31–40 years 11.9%, 41–50 years 12.9%, 51–60 years 16.4%, 61–70 years 18.7%, ≥70 years 26.7%. Odds of deficiency in the elderly (≥70 years) were 5.62 times higher than in children (0–18 years) (OR = 5.62, 95% CI: 5.29–5.97, *p* < 0.001).

### 3.5. Sex Differences

Females demonstrated slightly but significantly higher mean 25(OH)D compared to males (Table 5) (Mann–Whitney U = 1.36 × 10^9^, *p* < 0.001): females 62.6 ± 34.3 nmol/L (median: 60.0), males 61.0 ± 34.0 nmol/L (median: 58.0), difference 1.6 nmol/L (2.6%).

Vitamin D status distribution differed significantly between sexes (χ^2^ = 107.07, *df* = 3, *p* < 0.001). Females had slightly lower deficiency prevalence (15.4% vs. 16.7%, OR = 0.91, 95% CI: 0.88–0.94, *p* < 0.001) and higher optimal status prevalence (29.2% vs. 27.4%, OR = 1.09, 95% CI: 1.07–1.12, *p* < 0.001).

## 4. Discussion

### 4.1. Principal Findings

This large-scale retrospective analysis of 106,875 patients over a decade with valid 25OHD measurements revealed several key findings. First, we observed a substantial 14.6% increase in mean 25(OH)D concentrations during and after the COVID-19 pandemic compared to the pre-pandemic period, accompanied by 36% relative reduction in deficiency prevalence (from 19.6% to 12.5%). Second, pronounced seasonal variation persisted throughout the study period, with summer/autumn levels exceeding winter/spring by 21%. Third, vitamin D status declined progressively with age, with elderly patients (≥70 years) showing 19.4% lower levels and 4.4-fold higher deficiency prevalence compared to children. Finally, females demonstrated slightly higher vitamin D levels than males, with sex differences most pronounced during reproductive years.

### 4.2. Temporal Trends and COVID-19 Pandemic Effect

One of the principal observations in this study was that 25(OH)D concentrations were higher in the COVID/post-COVID period than in the pre-COVID period. However, because the dataset did not include information on vitamin D supplementation, dietary intake, body mass index, sun exposure, comorbidities, medications, COVID-19 status, or the clinical indication for testing, the reasons for this difference cannot be determined from the present data. The 8.4 nmol/L increase in mean 25(OH)D represents not only a statistically significant but also clinically meaningful improvement in vitamin D status among tested patients. This shift resulted in nearly one-third achieving optimal status (>75 nmol/L) in 2020–2023 compared to less than one-quarter in 2014–2019.

Several non-mutually exclusive explanations remain possible, including changes in supplementation behavior, healthcare-seeking patterns, physician testing practices, case mix, and broader public awareness related to vitamin D [36,37]. These should be regarded as hypotheses rather than conclusions directly supported by the dataset. Studies from various countries reported substantial increases in vitamin D supplement sales during 2020–2021 [38,39]. Second, healthcare providers may have been more proactive in recommending supplementation, particularly for high-risk patients [40]. Third, lockdown measures and work-from-home policies may have paradoxically increased recreational outdoor activities during permitted hours, potentially enhancing sun exposure in some demographic groups [41]. Fourth, the assay measures both 25(OH)D_2_ and 25(OH)D_3_, and the pandemic-era improvement reflects combined contributions from increased vitamin D_3_ supplementation and seasonal synthesis. The inability to differentiate vitamin D forms is a limitation of this analysis; future studies with vitamin D form-specific measurements would clarify the relative contribution of supplementation versus sun exposure to the observed improvement.

Importantly, 25(OH)D measurements were performed in an ISO 15189-accredited national reference laboratory that adheres to a national quality control program and multiple international external quality assessment schemes, with stable internal and external quality control performance throughout 2014–2023. These data make it highly unlikely that the observed temporal changes in vitamin D status are attributable to analytical drift rather than genuine population-level changes in 25(OH)D.

Findings on our Slovenian population align with recent reports showing improvements in vitamin D status during the pandemic. A UK study reported 31% increase in mean 25(OH)D between 2019 and 2020 [42], while Swiss researchers documented increased supplement use from 22% to 40% of the population [43]. Similarly, a multicenter European study found reduced deficiency prevalence from 40.1% to 29.3% between 2019 and 2021 [44].

These findings are further contextualized by population-level vitamin D supplementation trends. A comprehensive analysis of European supplement use patterns documented that retail sales of vitamin D products increased by 20–40% in 2020–2021 compared to the 2019 baseline [45], with the most substantial increases occurring in countries with the highest COVID-19 case numbers. This market-level data corroborates our laboratory-based findings of improved vitamin D status and suggests that increased supplementation was widespread rather than isolated to our Slovenian population. Furthermore, preliminary data from online search trends suggest sustained public interest in vitamin D beyond 2021, potentially explaining the persistent improvement observed in our 2023 data [46]. The confluence of increased public awareness, expanded healthcare provider recommendations, and sustained supplementation practices is consistent with both the initial pandemic-era improvement and its persistence through 2023, although alternative explanations related to changes in testing indications or patient mix cannot be excluded based on our data.

Importantly, all 25(OH)D measurements were performed in an ISO 15189-accredited national reference laboratory that follows a national quality control program and participates in multiple international EQA schemes for vitamin D. Stable internal and external quality control performance across 2014–2023 strongly argues against assay drift as an explanation for the observed temporal changes and supports the interpretation that these trends reflect genuine shifts in population vitamin D status.

Notably, improved vitamin D levels persisted through 2023, three years after the initial pandemic surge. This sustained improvement suggests pandemic-related changes in vitamin D supplementation and awareness may have resulted in lasting behavioral modifications rather than temporary responses [47].

### 4.3. Seasonal Variation: Implications for Public Health

The 11.8 nmol/L (21%) difference between summer/autumn and winter/spring represents substantial seasonal variation with important clinical implications. This variation persisted consistently across all study years and both COVID periods, indicating seasonal UVB exposure patterns remain the dominant driver despite increased supplementation.

A two-fold difference in deficiency prevalence between summer (10.4%) and winter (21.2%) suggests current supplementation practices are insufficient to fully compensate for reduced UVB-mediated synthesis during winter months. This has important implications for clinical practice and public health policy [48,49].

Observation that peak 25(OH)D occurred in August-September, approximately 2–3 months after peak solar radiation (June solstice), reflects the pharmacokinetics of vitamin D metabolism. Following UVB exposure, cutaneous vitamin D_3_ undergoes temperature-dependent isomerization and thermal conversion, followed by hepatic 25-hydroxylation, requiring 6–8 weeks to reach maximal circulating 25(OH)D [27].

Our findings support the rationale for season-specific vitamin D supplementation recommendations. Current guidelines typically provide year-round dosing (600–800 IU daily), but our data suggest winter-specific strategies (higher doses November–April) may be more effective for maintaining year-round sufficiency [50,51]. Several studies demonstrated intermittent high-dose supplementation during winter effectively prevents seasonal decline [52,53].

### 4.4. Age-Related Decline

Progressive decline from 68.4 nmol/L in children to 55.1 nmol/L in older adults reflects multiple physiological changes. Aging is associated with reduced capacity for cutaneous synthesis due to decreased 7-dehydrocholesterol (approximately 75% reduction by age 70) [54]; decreased renal 1α-hydroxylase activity [55]; reduced intestinal absorption [56]; decreased outdoor activity [57]; changes in body composition with increased adipose tissue sequestering vitamin D [58]; and medication interactions [59]. Beyond volumetric dilution and sequestration in adipose tissue, obesity has also been linked to reduced hepatic 25-hydroxylase activity, which lowers 25(OH)D production and thereby limits substrate availability for local activation in target tissues [60]. This mechanism may contribute to the higher prevalence of vitamin D deficiency observed in individuals with obesity.

The 4.4-fold higher deficiency prevalence in the elderly (26.7%) compared to children (6.1%) has important clinical implications. Vitamin D deficiency in older adults is associated with increased risk of falls, fractures, sarcopenia, cognitive decline, and all-cause mortality [61,62,63]. Moreover, the elderly often have concurrent conditions, increasing vitamin D requirements, including chronic kidney disease, malabsorption syndromes, and osteoporosis [64].

Relatively high vitamin D in children may reflect widespread supplementation practices in infancy/early childhood recommended by pediatric societies [65], fortification of infant formula and dairy products, higher outdoor activity, and more efficient cutaneous synthesis. However, 6.1% deficiency prevalence indicates supplementation and fortification remain suboptimal for some children.

Our findings support the development of age-specific guidelines. While children and younger adults may achieve adequate status with modest supplementation (400–600 IU daily), the elderly likely require higher doses (800–2000 IU daily) to maintain optimal 25(OH)D, particularly during winter [66]. Routine screening of elderly patients, especially those with limited mobility or institutionalized, should be considered.

### 4.5. Sex Differences

The 1.6 nmol/L (2.6%) higher vitamin D in females, while statistically significant, represents a relatively modest difference with limited clinical impact. Although the absolute difference in mean 25(OH)D between sexes (1.6 nmol/L) is small and within the range of analytical and biological variability for this assay, the consistency of slightly higher values in females across age groups suggests a modest but systematic tendency rather than a purely random phenomenon. However, consistency across age groups and seasons suggests genuine biological or behavioral differences.

Several mechanisms may contribute. Estrogen influences vitamin D metabolism, potentially enhancing 25-hydroxylation efficiency [67]. Sex differences in body composition may affect distribution [68]. Genetic polymorphisms in vitamin D-related genes show sex-specific effects in some populations [69]. Women may be more likely to use vitamin D supplements as part of calcium supplementation for bone health [70]. Differences in healthcare-seeking behavior exist, with women more likely to undergo preventive screening [71].

Although females had slightly higher 25(OH)D concentrations than males overall, the magnitude of the sex difference across age groups was modest, and the descriptive patterns in Figure 4 should not be overinterpreted. These data support a small overall sex-related difference among tested individuals, but they do not allow firm conclusions about age-specific hormonal or behavioral mechanisms [72].

### 4.6. Strengths and Limitations

Strengths: This study has several notable strengths. First, an exceptionally large dataset (107,900 records, including 106,875 patients with valid 25OHD measurements) provided robust statistical power for detecting clinically meaningful differences and enabled detailed subgroup analyses. Second, a decade-long study period allowed examination of temporal trends and captured a major public health event (COVID-19 pandemic). Third, near-complete data for vitamin D (99.1%) minimized selection bias. Fourth, a diverse age range (0–119 years) enabled comprehensive age-stratified analyses. Fifth, inclusion of patients from routine clinical practice enhances generalizability compared to highly selected research cohorts. Sixth, the study was conducted in a national reference laboratory with rigorously maintained analytical quality: continuous internal quality control (IQC) at three concentration levels with coefficients of variation approximately 3–7%, participation in national and international external quality assessment (EQA) schemes including DEQAS (Vitamin D External Quality Assessment Scheme) and other IFCC-endorsed programs, and ISO 15189 accreditation for medical laboratory quality. This robust quality framework, together with the use of the same Architect analyzer and assay over the entire 10-year period, minimizes the likelihood that the reported temporal trends are due to analytical drift and ensures that observed improvements in vitamin D status reflect genuine population-level changes.

A strength of this study is our comprehensive data quality assessment. We identified and excluded 225 records (0.21%) with implausible values: 180 with age > 100 years and 45 with vitamin D > 150 nmol/L. These exclusions, representing < 0.2% of the dataset, resulted in negligible changes to descriptive statistics, enhancing confidence in study findings.

Limitations: Our dataset reflects individuals referred for vitamin D testing in routine care and is therefore vulnerable to selection bias. The results should be interpreted as patterns among tested patients rather than as direct estimates for the general population. Because the indications for 25(OH)D testing were not uniformly recorded and may have changed over time, especially during and after 2020, the observed temporal patterns should be interpreted as descriptive trends among tested individuals rather than direct evidence of changes in vitamin D status in the general Slovenian population. This limitation also applies to neonates and infants. In these age groups, 25(OH)D testing in routine hospital practice is likely to have reflected specific physician-directed clinical concerns rather than general population screening, but the retrospective dataset did not contain standardized indication codes to verify the exact reason for testing in each case. The present study was not designed to evaluate temporal trends in potentially toxic 25(OH)D concentrations. Values > 150 nmol/L were excluded during data cleaning because they were considered biologically implausible in the context of the retrieved routine laboratory dataset and were consistent with likely data-entry error in the most extreme cases; therefore, post-2020 trends in high-end or potentially toxic values could not be analyzed within the final cohort.

Several limitations warrant consideration. First, our analysis is based on clinically indicated vitamin D testing rather than population-based sampling. Indications for 25(OH)D measurement may have changed over time, especially during the COVID-19 pandemic, when both public and professional interest in vitamin D increased substantially. Because clinical indications for testing (e.g., osteoporosis assessment, malabsorption, suspected deficiency, and acute COVID-19 illness) were not uniformly recorded in our laboratory information system, we could not adjust for potential selection bias related to changing testing indications. Thus, our findings should be interpreted as trends among patients tested in routine clinical care, rather than as definitive estimates of population prevalence. Future studies using population-based sampling or linkage with prescription and diagnosis registries are needed to disentangle behavioral changes (e.g., increased supplementation) from shifts in testing indications. Second, as a retrospective observational study, we cannot infer causality regarding temporal trends. While we hypothesize that increased vitamin D supplementation drove improvements during the COVID-19 period, we lacked data on supplement use, dietary intake, or sun exposure behaviors. Third, we included only the first measurements for each patient, precluding within-individual longitudinal analysis. Fourth, we lacked clinical data on comorbidities, medications, body mass index, or specific indications for testing, limiting our ability to adjust for confounding. Fifth, a single geographic region limits generalizability to populations at different latitudes or with different demographics. Sixth, although we cannot absolutely exclude minor long-term shifts in assay calibration, the documented IQC and EQA performance over 2014–2023 makes substantial analytical drift an unlikely explanation for the observed temporal trends. Seventh, we cannot distinguish between vitamin D_2_ and D_3_, which may have different efficacy [73]. Eighth, seasonal classifications based on calendar months may not perfectly align with actual UVB exposure, which varies by weather, geographic microclimates, and individual behaviors. Ninth, the study did not account for pre-analytical factors that may influence vitamin D measurements, including specimen collection time, storage conditions, or seasonal variation in testing time-of-day. In addition, vitamin D status categories depend on the threshold system applied. Because alternative clinical cutoffs are used in the literature, the prevalence of deficiency, insufficiency, and sufficiency reported here should be interpreted within the context of the selected classification framework. These factors could introduce minor variation in reported concentrations.

In addition, the laboratory database does not reliably capture clinical indications or diagnoses for each vitamin D request. Consequently, we could not stratify results by underlying health condition, including acute COVID-19 illness, and we cannot exclude that temporal changes in the mix of clinical indications may have contributed to some of the observed patterns.

Ethnicity and skin pigmentation are well-recognized determinants of vitamin D status, with individuals of darker skin often exhibiting lower 25(OH)D at similar UVB exposure. Our dataset, derived from a predominantly white European population in Slovenia, does not include ethnicity data, and we were therefore unable to evaluate ethnic differences. This limits the generalizability of our findings to more ethnically diverse populations.

An important contextual consideration is that lockdown stringency and duration varied considerably across Europe during the 2020–2023 period. During 2020–2021, Slovenia implemented multiple waves of public health restrictions, including stay-at-home orders, closures of non-essential services, limits on social gatherings, and intermittent school and workplace closures [74]. Our data represent a single geographic region and may not generalize to countries with substantially different COVID-19 policies or climatic patterns. Future studies examining vitamin D trends across multiple European centers with documented lockdown stringency levels would strengthen causal inference regarding whether the observed shift may reflect multiple factors and their relationship to public health interventions.

### 4.7. Clinical and Public Health Implications

Our findings suggest several potential clinical implications that warrant evaluation in future intervention studies: (1) In Slovenia, where approximately one quarter of tested adults ≥ 70 years had 25(OH)D < 30 nmol/L, targeted case-finding or systematic screening strategies for high-risk older adults could be evaluated. (2) Season-specific vitamin D supplementation strategies, with higher doses during November–April, may help prevent seasonal decline, particularly in elderly or homebound patients, although optimal dosing protocols require prospective validation. (3) Age-appropriate dosing recommendations, with elderly individuals potentially requiring 800–2000 IU daily to maintain adequate status, should be tested in randomized trials. (4) Sustained public education about vitamin D beyond pandemic contexts may help maintain the observed improvements. (5) Continued monitoring will be essential to determine whether improved status persists or regresses in the coming years. At the public health level, our findings support ongoing discussions about: (1) vitamin D supplementation recommendations for high-risk groups (elderly, institutionalized, those with limited outdoor exposure); (2) expanded food fortification programs in Central/Eastern Europe, particularly to mitigate winter and late-life deficiency, although we did not assess the effectiveness, safety, or cost-effectiveness of specific fortification strategies; (3) seasonal health campaigns promoting awareness during autumn–winter months; and (4) integration of vitamin D assessment into routine geriatric care. However, these policy recommendations require evaluation through implementation research and cost-effectiveness analysis before widespread adoption.

## 5. Conclusions

In this large retrospective laboratory-based cohort of tested individuals, serum 25(OH)D concentrations were higher in 2020–2023 than in 2014–2019, while substantial seasonal and age-related differences persisted. These findings support continued monitoring of vitamin D status in clinical practice, especially in older adults and during winter, but they do not establish causal effects of the pandemic or provide population-representative prevalence estimates. The study identifies persistent seasonal and age-related gradients and a higher concentration profile in the 2020–2023 period among tested individuals. Further studies integrating laboratory, clinical, and prescription data are required to determine the mechanisms underlying these trends and to evaluate whether similar patterns are present at the population level.

### Future Directions and Research Implications

Several questions remain unanswered by this observational analysis. First, prospective studies incorporating individual-level data on supplement use, dietary intake, sunscreen use, and outdoor exposure would clarify the relative contributions of supplementation versus behavioral factors to the observed pandemic-era improvements. Second, the inability to distinguish between vitamin D2 and D3 in our assay precluded the determination of whether the pandemic-era improvement reflected supplementation-driven D3 increases or enhanced endogenous D2-derived supplementation. Future studies employing form-specific vitamin D measurements would address this gap. Third, investigation of whether sustained pandemic-era behavioral changes correspond to improved clinical outcomes related to bone health, infection risk, and respiratory disease would strengthen the public health relevance of our findings. Fourth, examination of whether the pandemic-era shift in population vitamin D status persists post-2023 or regresses would determine whether public health messaging effects are sustainable or require ongoing reinforcement.

Future studies linking laboratory data with clinical records, prescription data, and diagnosis registries should apply multivariable and interrupted time-series methods to better assess whether temporal changes persist after adjustment for age, sex, seasonality, testing indication, and comorbidity burden.

## Figures and Tables

**Table 1:** Baseline characteristics of core analytic cohort (*n* = 106,875 patients with valid 25(OH)D measurements).

Characteristic	Overall (*n* = 106,875)
Age (years), mean ± SD	47.7 ± 27.6
Age range (min–max)	0.0–100
Female, *n* (%)	60,913 (56.5%)
Male, *n* (%)	46,987 (43.5%)
25(OH)D (nmol/L), mean ± SD	61.9 ± 34.2
25(OH)D (nmol/L), median (IQR)	59.0 (39.0–79.0)
25(OH)D range (min–max)	1.0–150.0
Deficient (<30 nmol/L), *n* (%)	17,059 (16.0%)
Insufficient (30–50 nmol/L), *n* (%)	24,354 (22.8%)
Adequate (50–75 nmol/L), *n* (%)	35,096 (32.8%)
Optimal (>75 nmol/L), *n* (%)	30,366 (28.4%)
Severe deficiency (<12.5 nmol/L), *n* (%)	1247 (1.2%)
Measurement completion rate, %	99.1%

**Table 2:** Comparison of vitamin D levels and status: pre-COVID versus COVID/post-COVID periods.

Characteristic	Pre-COVID (2014–2019)	COVID/Post-COVID (2020–2023)	*p*-Value
*n*	52,435	55,465	-
Age (years), mean ± SD	48.2 ± 27.9	47.2 ± 27.4	<0.001
Female, *n* (%)	29,548 (56.4%)	31,365 (56.5%)	>0.05
Mean ± SD (nmol/L)	57.6 ± 34.0	66.0 ± 33.8	<0.001 *
Median (IQR) (nmol/L)	54.0 (35.0–74.0)	63.0 (44.0–83.0)	<0.001 *
Deficient (<30 nmol/L), *n* (%)	10,163 (19.6%)	6896 (12.5%)	<0.001 †
Insufficient (30–50 nmol/L), *n* (%)	13,051 (25.2%)	11,303 (20.5%)	<0.001 †
Adequate (50–75 nmol/L), *n* (%)	16,475 (31.8%)	18,621 (33.8%)	<0.001 †
Optimal (>75 nmol/L), *n* (%)	12,116 (23.4%)	18,250 (33.1%)	<0.001 †

* Mann–Whitney U test; † Chi-square test (χ^2^ = 2023.58, *df* = 3).

**Table 3:** Seasonal variation in vitamin D levels and deficiency prevalence.

Season	*n*	Mean ± SD (nmol/L)	Median (IQR)	Deficient < 30, *n* (%)	Insufficient 30–50, *n* (%)	Adequate 50–75, *n* (%)	Optimal > 75, *n* (%)
Winter	27,700	56.5 ± 33.1	52.0 (33.0–73.0)	5884 (21.2%)	7279 (26.3%)	8306 (30.0%)	6231 (22.5%)
Spring	28,383	56.3 ± 35.1	52.0 (34.0–72.0)	5970 (21.0%)	7557 (26.6%)	8764 (30.9%)	6092 (21.5%)
Summer	20,253	68.0 ± 33.7	65.0 (47.0–84.0)	2102 (10.4%)	3842 (19.0%)	7187 (35.5%)	7122 (35.2%)
Autumn	30,539	68.1 ± 32.8	65.0 (47.0–84.0)	3103 (10.2%)	5676 (18.6%)	10,839 (35.5%)	10,921 (35.8%)

Statistical test: Kruskal–Wallis H = 4750.27, *p* < 0.001; Chi-square test (χ^2^ = 2874.36, *p* < 0.001).

**Table 4:** Vitamin D levels and deficiency prevalence by age group.

Age Group (Years)	*n*	Mean ± SD (nmol/L)	Median (IQR) (nmol/L)	Female, *n* (%)	Deficient < 30, *n* (%)
0–18	25,434	68.4 ± 30.7	64.0 (49.0–83.0)	12,767 (50.1%)	1543 (6.1%)
19–30	8443	63.5 ± 33.8	60.0 (42.0–78.0)	5173 (61.1%)	959 (11.4%)
31–40	8475	65.7 ± 38.5	61.0 (42.0–81.0)	5679 (66.8%)	1006 (11.9%)
41–50	9239	64.2 ± 41.6	59.0 (41.0–79.0)	5500 (59.2%)	1194 (12.9%)
51–60	11,914	61.6 ± 37.5	58.0 (38.0–78.0)	6904 (57.5%)	1953 (16.4%)
61–70	14,793	59.9 ± 31.6	57.0 (36.0–78.0)	7911 (52.9%)	2766 (18.7%)
≥70	28,562	55.1 ± 31.5	52.0 (29.0–75.0)	16,978 (58.2%)	7638 (26.7%)

Statistical test: Kruskal–Wallis H = 2944.40, *p* < 0.001; Chi-square test for trend: χ^2^ = 3856.72, *p* < 0.001.

**Table 5:** Comparison of vitamin D levels and status between males and females.

Characteristic	Male	Female	*p*-Value
*n*	46,987	60,913	-
Age (years), mean ± SD	46.4 ± 28.2	48.7 ± 27.2	<0.001
Age (years), median	46.0	50.0	<0.001
Mean ± SD (nmol/L)	61.0 ± 34.0	62.6 ± 34.3	<0.001 *
Median (IQR) (nmol/L)	58.0 (38.0–78.0)	60.0 (40.0–79.0)	<0.001 *
Deficient (<30 nmol/L), *n* (%)	7779 (16.7%)	9280 (15.4%)	<0.001 †
Insufficient (30–50 nmol/L), *n* (%)	11,076 (23.8%)	13,278 (22.0%)	<0.001 †
Adequate (50–75 nmol/L), *n* (%)	14,960 (32.1%)	20,136 (33.4%)	<0.001 †
Optimal (>75 nmol/L), *n* (%)	12,760 (27.4%)	17,606 (29.2%)	<0.001 †

* Mann–Whitney U test; † Chi-square test (χ^2^ = 107.07, *df* = 3, *p* < 0.001)

## Data Availability

The data that support the findings of this study are available from University Medical Center Ljubljana, but restrictions apply to the availability of these data, which were used under license for the current study, and so are not publicly available. Data are, however, available from the authors upon reasonable request and with permission of the University Medical Center Ljubljana.

## Supplementary Materials

The following supporting information can be downloaded at: https://www.mdpi.com/article/10.3390/nu18071168/s1, Supplementary Table S1: Annual trends in vitamin D levels and status (2014–2023); Supplementary Table S2: Monthly variation in vitamin D levels and deficiency prevalence; Supplementary Table S3: Internal and external quality control performance data.
