# Supplementary material for: Ten-Year Trends in Serum 25-Hydroxyvitamin D in Slovenia (2014–2023): Laboratory-Based Data from Tested Individuals and COVID-19-Period Changes
Source: Nutrients. 2026 Apr 7;18(7):1168. doi: 10.3390/nu18071168 (PMC13074350; doi:10.3390/nu18071168)
Supplement: Supplementary file 1 [file nutrients-18-01168-s001.zip › nutrients-4227644-supplementary.pdf]

Supplementary Materials

The following supporting information can be downloaded at the journal website: Supplementary Table S1: Annual trends in vitamin D levels and status (2014-2023); Supplementary Table S2: Monthly variation in vitamin D levels and deficiency prevalence; Supplementary Table S3: Internal and external quality control performance data.

Supplementary Table S1. Annual trends in vitamin D levels and status (2014-2023).

Year	N	Mean ± SD (nmol/L)	Median (IQR) (nmol/L)	Deficient <30, n (%)	Optimal >75, n (%)
2014	6,241	57.0 ± 26.4	54.0 (37.0-72.0)	925 (14.9%)	1,334 (21.5%)
2015	6,775	55.1 ± 28.2	52.0 (35.0-70.0)	1,253 (18.5%)	1,312 (19.4%)
2016	8,493	57.9 ± 39.3	54.0 (32.0-76.0)	1,935 (22.8%)	2,155 (25.4%)
2017	10,463	57.4 ± 35.5	54.0 (34.0-74.0)	2,187 (20.9%)	2,444 (23.4%)
2018	8,231	60.0 ± 37.2	57.0 (37.0-76.0)	1,515 (18.4%)	2,121 (25.8%)
2019	9,878	57.9 ± 32.0	55.0 (36.0-74.0)	1,941 (19.6%)	2,353 (23.8%)
2020	11,030	64.8 ± 35.8	62.0 (42.0-81.0)	1,553 (14.1%)	3,497 (31.7%)
2021	16,518	65.4 ± 34.0	62.0 (43.0-82.0)	2,099 (12.7%)	5,276 (31.9%)
2022	14,738	65.3 ± 33.0	62.0 (43.0-83.0)	1,975 (13.4%)	4,836 (32.8%)
2023	14,538	67.2 ± 33.2	64.0 (45.0-84.0)	1,676 (11.5%)	5,038 (34.7%)

Statistical test: Kendall's $\tau = 0.093$, $p < 0.001$

Supplementary Table S2. Monthly variation in vitamin D levels and deficiency prevalence.

Month	N	Mean ± SD (nmol/L)	Median (nmol/L)	Deficient <30, n (%)
January	10,229	56.5 ± 33.1	52.0	2,220 (21.7%)
February	8,075	54.8 ± 35.2	50.0	1,998 (24.7%)
March	10,202	55.6 ± 37.2	51.0	2,445 (24.0%)
April	8,154	54.4 ± 35.0	50.0	1,912 (23.4%)
May	10,027	58.6 ± 32.6	55.0	1,613 (16.1%)
June	8,642	62.2 ± 30.5	60.0	956 (11.1%)
July	6,070	69.7 ± 33.2	67.0	645 (10.6%)
August	5,541	75.0 ± 37.2	72.0	501 (9.0%)
September	9,367	73.7 ± 33.1	71.0	739 (7.9%)
October	10,417	68.2 ± 32.1	66.0	1,043 (10.0%)
November	10,755	63.2 ± 32.6	60.0	1,321 (12.3%)
December	9,396	57.9 ± 31.0	55.0	1,666 (17.7%)

Supplementary Table S3: QC - Annual Summary (2014-2023)

Year	IQC CV% Low (~30 nmol/L)	IQC CV% Mid (~60 nmol/L)	IQC CV% High (~80 nmol/L)	INSTAND Bias%	Within limits (%)
**2014**	6.8	5.4	4.2	5.2	96.7
**2015**	6.5	5.2	4.0	4.8	97.2
**2016**	6.2	5.0	3.9	3.9	97.8
**2017**	5.9	4.8	3.8	4.2	98.1
**2018**	5.8	4.7	3.7	3.5	98.5
**2019**	5.6	4.5	3.6	2.8	98.8
**2020**	5.4	4.4	3.5	3.1	99.0

**2021**	5.3	4.3	3.4	2.4	99.2
**2022**	5.2	4.2	3.4	2.7	99.1
**2023**	5.1	4.1	3.3	2.1	99.3
